# Genome sequence of *Bradyrhizobium* sp. WSM1253; a microsymbiont of *Ornithopus compressus* from the Greek Island of Sifnos

**DOI:** 10.1186/s40793-015-0115-9

**Published:** 2015-11-30

**Authors:** Ravi Tiwari, John Howieson, Ron Yates, Rui Tian, Britanny Held, Roxanne Tapia, Cliff Han, Rekha Seshadri, T. B. K. Reddy, Marcel Huntemann, Amrita Pati, Tanja Woyke, Victor Markowitz, Natalia Ivanova, Nikos Kyrpides, Wayne Reeve

**Affiliations:** Centre for Rhizobium Studies, Murdoch University, Murdoch, Australia; Department of Agriculture and Food, South Perth, Australia; Los Alamos National Laboratory, Bioscience Division, Los Alamos, NM USA; DOE Joint Genome Institute, Walnut Creek, CA USA; Biological Data Management and Technology Center, Lawrence Berkeley National Laboratory, Berkeley, CA USA; Department of Biological Sciences, King Abdulaziz University, Jeddah, Saudi Arabia

**Keywords:** root-nodule bacteria, nitrogen fixation, rhizobia, *Ornithopus*

## Abstract

*Bradyrhizobium* sp. WSM1253 is a novel N_2_-fixing bacterium isolated from a root nodule of the herbaceous annual legume *Ornithopus compressus* that was growing on the Greek Island of Sifnos. WSM1253 emerged as a strain of interest in an Australian program that was selecting inoculant quality bradyrhizobial strains for inoculation of Mediterranean species of lupins (*Lupinus angustifolius, L. princei, L. atlanticus, L. pilosus*). In this report we describe, for the first time, the genome sequence information and annotation of this legume microsymbiont. The 8,719,808 bp genome has a G + C content of 63.09 % with 71 contigs arranged into two scaffolds. The assembled genome contains 8,432 protein-coding genes, 66 RNA genes and a single rRNA operon. This improved-high-quality draft rhizobial genome is one of 20 sequenced through a DOE Joint Genome Institute 2010 Community Sequencing Project.

**Electronic supplementary material:**

The online version of this article (doi:10.1186/s40793-015-0115-9) contains supplementary material, which is available to authorized users.

## Introduction

Root nodule bacteria are soil microorganisms that can establish a symbiotic relationship with hosts from the legume plant family *Leguminosae.* In this intimate relationship the bacteria fix atmospheric nitrogen into ammonia for the legume, in exchange for nutrients. With the continued discovery of a large number of organisms with this capability through the last century, the slow growing, non-acid producing root nodule bacteria were separated from the fast growing acid-producing forms and designated the bradyrhizobia [1]. The initial interest in the bradyrhizobia arose from the ability of strains to nodulate agriculturally important crops such as soybean and groundnut. Today the bradyrhizobia are known to nodulate a wide variety of legumes such as *Arachis hypogaea**, Adenocarpus spp.,**Beta vulgaris**, Chamaecytisus spp.,**Cytisus villosus**, Entada koshunensis, Glycine spp.,**Dolichos lablab**, Lespedeza* spp., *Lupinus spp., Ornithopus* spp*.,**Pachyrhizus erosus*, *Spartocytisus spp.* and *Teline* spp. [2–9].

Two agriculturally important legume genera form a symbiosis with *Bradyhizobium* [10]*,* the subject of this manuscript. *Lupinus* which is a large and diverse genus, and *Ornithopus**,* which is a smaller forage legume genus, both nodulate and fix nitrogen with this bacterium. *Lupinus angustifolius* is commonly known as lupin in Europe and Australia, and lupine in North America, and its grain is widely used as an animal or human food. Lupins are either annual or perennial herbs, shrubs or trees [11]. *Ornithopus* is commonly known as serradella, and was originally confined to the Iberian peninsula and the Mediterranean basin, however it has become a valuable grazing plant adapted to low rainfall, acidic and infertile soils world-wide [12]. Hence, appropriate *Bradyrhizobium* inoculants are of particular value for the establishment of effective nitrogen-fixing symbioses with these legume genera.

In Australia, the challenge was to select inoculant strains that were optimal for N fixation in symbiosis with *Lupinus angustifolius* and several species of *Ornithopus**.* These are all very important legumes in farming systems of Western Australia. They are cultivated on the same acid and sandy soils, and share microsymbionts [13]. Thus, it was important that any inoculant strain released for an individual legume species did not compromise the potential nitrogen fixation from the other legumes. *Bradyrhizobium sp.*WSM1253 emerged as a strain of interest in an Australian program that was selecting inoculant strains for Mediterranean species of lupins*.* Strain WSM1253 was isolated from a nodule of the herbaceous annual legume *Ornithopus compressus* in 1991 collected 2.5 km near of Kastro, towards Faros, on the Greek Island of Sifnos. This strain was found to be capable of high levels of nitrogen fixation across many species in the cross-nodulation complex of lupins and *Ornithopus**,* being particularly effective on *L. princei* [14]. Here we present a preliminary description of the general features of the *Ornithopus compressus* microsymbiont *Bradyrhizobium* sp. WSM1253, together with the description of the complete genome sequence and its annotation.

## Organism information

### Classification and features

**Fig. 1:**
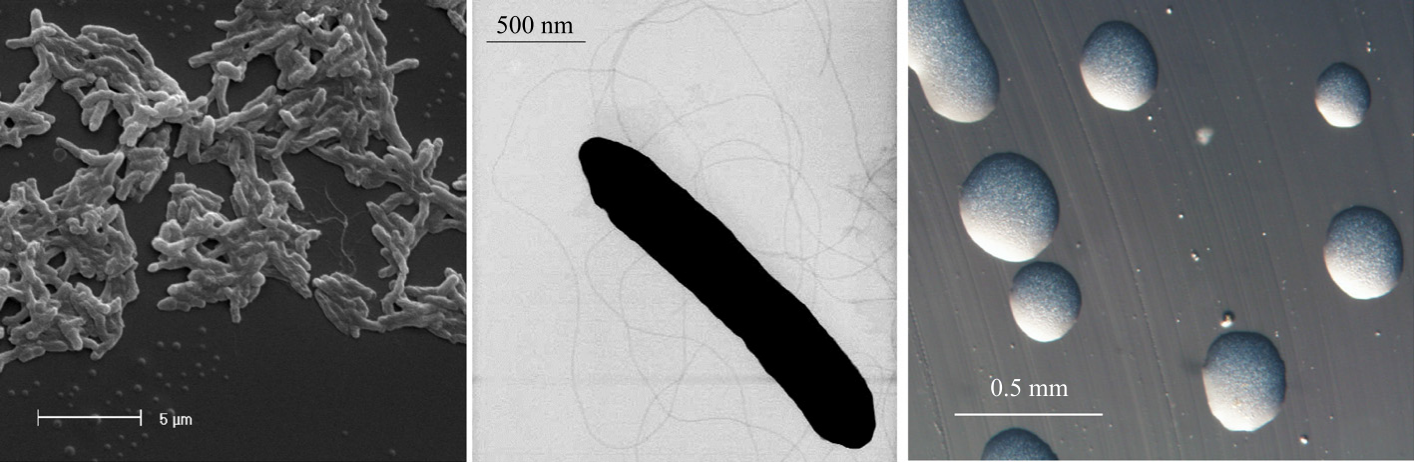
Images of *Bradyrhizobium* sp. WSM1253 using scanning (Left) and transmission (Center) electron microscopy as well as light microscopy to visualize colony morphology on solid media (Right)

*Bradyrhizobium* sp. WSM1253 is a motile, non-sporulating, non-encapsulated, Gram-negative rod in the order *Rhizobiales* of the class *Alphaproteobacteria*. The rod shaped form varies in size and dimensions of approximately 0.25 μm in width and 1.5-2.0 μm in length (Fig. 1 Left and Center). It is relatively slow growing, forming colonies after 6–7 days when grown on ½LA [15], TY [16] or YMA [17] at 28 °C. Colonies on ½LA are opaque, slightly domed and moderately mucoid with smooth margins (Fig. 1 Right).

**Fig. 2:**
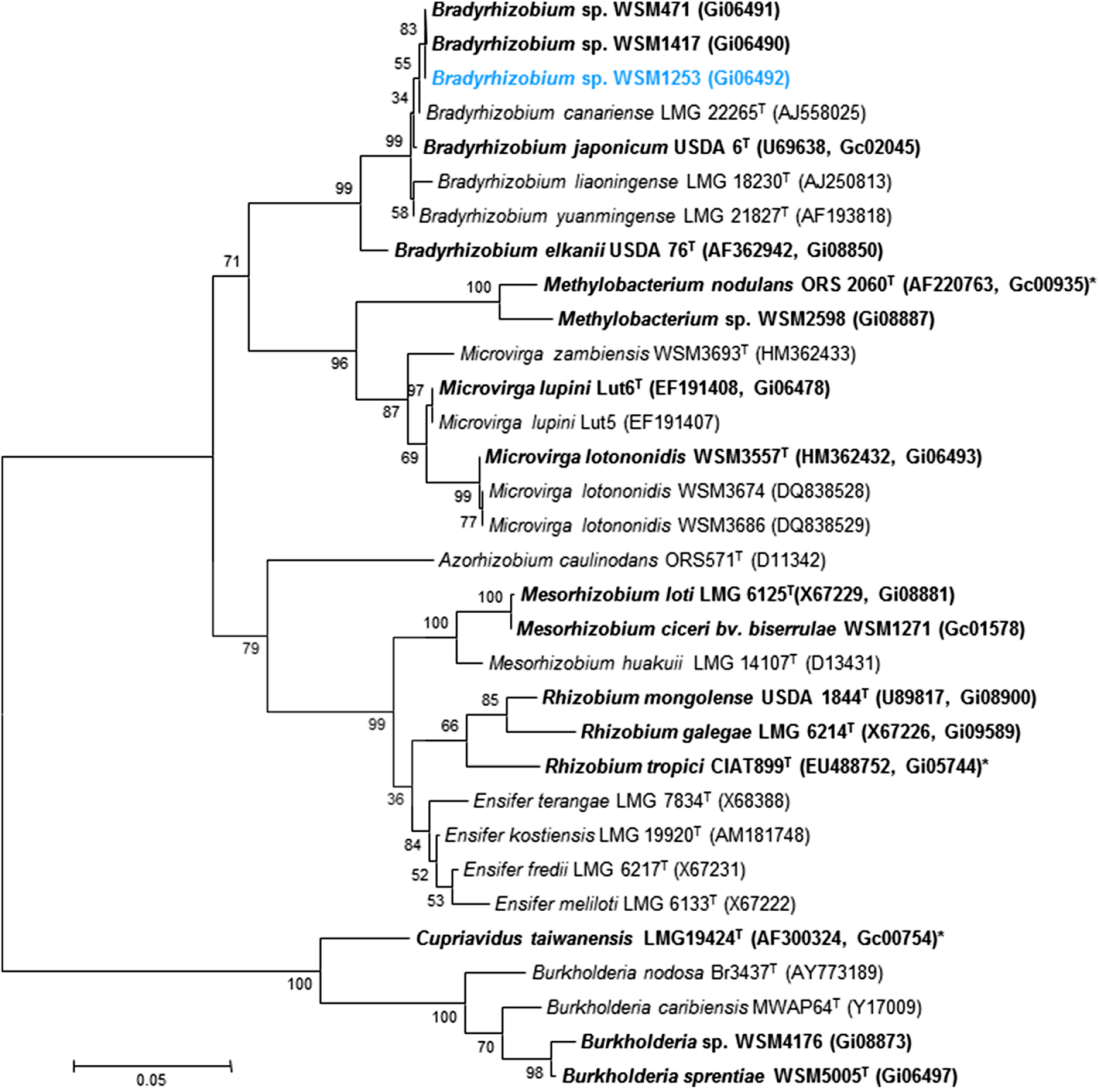
Phylogenetic tree showing the relationship of *Bradyrhizobium* sp. WSM1253 (shown in bold print) to other root nodule bacteria based on aligned sequences of a 1,012 bp internal region the 16S rRNA gene. All sites were informative and there were no gap-containing sites. Phylogenetic analyses were performed using MEGA [41], version 5. The tree was built using the Maximum-Likelihood method with the General Time Reversible model [42]. Bootstrap analysis [43] with 500 replicates was performed to assess the support of the clusters. Type strains are indicated with a superscript T. Brackets after the strain name contains a DNA database accession number and/or a GOLD ID (beginning with the prefix G) for a sequencing project registered in GOLD [22]. Published genomes are indicated with an asterisk

Minimum Information about the Genome Sequence (MIGS) is provided in Table 1 and Additional file 1: Table S1. Strain WSM1253 shares 100 % (1369/1369 bp), 99.85 % (1367/1369 bp) and 99.48 % (1362/1369 bp) 16S rRNA sequence identity with *Bradyrhizobium* sp. WSM1417, *Bradyrhizobium* sp. BTA-1^T^ and *Bradyrhizobium japonicum*USDA 6^T^*,* respectively as determined using NCBI BLAST analysis [18]. Figure 2 shows the phylogenetic neighbor-hood of *Bradyrhizobium* sp. WSM1253 in a 16S rRNA sequence based tree.

**Table 1:** Classification and general features of *Bradyrhizobium* sp. WSM1253 [44, 45]

MIGS ID	Property	Term	Evidence code^a^
	Classification	Domain *Bacteria*	TAS [45]
		Phylum *Proteobacteria*	TAS [46]
		Class *Alphaproteobacteria*	TAS [47, 48]
		Order *Rhizobiales*	TAS [49]
		Family *Bradyrhizobiaceae*	TAS [50, 51]
		Genus *Bradyrhizobium*	TAS [1]
		Species sp.	IDA
		Strain: WSM1253	TAS [14]
	Gram stain	Negative	IDA
	Cell shape	Rod	IDA
	Motility	Motile	IDA
	Sporulation	Non-sporulating	NAS
	Temperature range	Mesophile	NAS
	Optimum temperature	28 °C	NAS
	pH range; Optimum	5-9; 7	NAS
	Carbon source	Varied	IDA
MIGS-6	Habitat	Soil, root nodule, on plant host	TAS [14]
MIGS-6.3	Salinity	Non-halophilie	NAS
MIGS-22	Oxygen requirement	Aerobic	TAS [14]
MIGS-15	Biotic relationship	free-living, symbiont	TAS [14]
MIGS-14	Pathogenicity	Non-pathogenic	NAS
MIGS-4	Geographic location	Greek Island of Sifnos	TAS [14]
MIGS-5	Nodule collection date	1991	IDA
MIGS-4.1	Latitude	39.975	IDA
MIGS-4.2	Longitude	24.743889	IDA
MIGS-4.4	Altitude	Not reported	IDA

^a^Evidence codes – IDA: Inferred from Direct Assay; TAS: Traceable Author Statement (i.e., a direct report exists in the literature); NAS: Non-traceable Author Statement (i.e., not directly observed for the living, isolated sample, but based on a generally accepted property for the species, or anecdotal evidence). These evidence codes are from the Gene Ontology project [52]

#### Symbiotaxonomy

Few of the legumes of the Mediterranean basin introduced to agriculture elsewhere are nodulated by bacteria in the genus *Bradyrhizobium* [19]*.* Amongst the notable exceptions are *Lupinus* and *Ornithopus**,* which are legume genera adapted specifically to conditions of acidity and infertility [20]. Further, these two quite different legumes share a common species of *Bradyrhizobium**,* although their modes of infection and nodule structure differ substantially [21]. WSM1253 is unusual in being a highly effective microsymbiont for many species in the two legume genera discussed, including, *L. angustifolius, L. princei, L. atlanticus, L. pilosus, O. compressus, O. sativus* Brot. and *O. pinnatus* (Table 2). WSM1253 will therefore be a valuable strain to study the genetics of nodulation and nitrogen fixation in legumes of vastly differing physiology.

**Table 2:** Compatibility of *Bradyrhizobium* sp. WSM1253 [14] with different wild and cultivated legume species

Species name	Family	Common name	Habit/Growth type	Nod	Fix
*Lupinus atlanticus*	Fabaceae	Atlas Lupin/Moroccan Lupin	Annual herbaceous	+	+
*Lupinus pilosus*	Fabaceae	Mountain blue lupin	Annual herbaceous	+	+
*Lupinus princei*	Fabaceae	Lupin	Annual herbaceous	+	+
*Ornithopus pinnatus*	Fabaceae	Sand Bird’s-foot	Annual herbaceous	+	+
*Ornithopus sativus* Brot.	Fabaceae	common bird’s-foot	Annual herbaceous	+	+
*Ornithopus compressus*	Fabaceae	Yellow serradella	Annual herbaceous	+	+

+, nodulation/fixation observed

## Genome sequencing information

### Genome project history

This organism was selected for sequencing on the basis of its environmental and agricultural relevance to issues in global carbon cycling, alternative energy production, and biogeochemical importance, and is part of the Community Sequencing Program at the U.S. Department of Energy, Joint Genome Institute for projects of relevance to agency missions. The genome project is deposited in the Genomes OnLine Database [22] and the improved-high-quality draft genome sequence in IMG. Sequencing, finishing and annotation were performed by the JGI. A summary of the project information is shown in Table 3.

**Table 3:** Project information

MIGS ID	Property	Term
MIGS 31	Finishing quality	Improved-high-quality draft
MIGS 28	Libraries used	Illumina GAii and 454 FLX libraries
MIGS 29	Sequencing platforms	Illumina and 454
MIGS 31.2	Fold coverage	659.4 × Illumina; 8.4 × 454
MIGS 30	Assemblers	Velvet 1.0.13; Newbler 2.3
MIGS 32	Gene calling methods	Prodigal 1.4
	Locus Tag	Bra1253
	GenBank ID	AHMB01000000
	Genbank Date of Release	May 4, 2012
	GOLD ID	Gp0007394
	BIOPROJECT	PRJNA62341
MIGS 13	Project relevance	Symbiotic N2 fixation, agriculture
	Source Material Identifier	WSM1253

### Growth conditions and genomic DNA preparation

*Bradyrhizobium* sp. WSM1253 was grown on TY solid medium for 10 days, a single colony was selected and used to inoculate 5 ml TY broth medium. The culture was grown for 96 h on a gyratory shaker (200 rpm) at 28 °C [23]. Subsequently 1 ml was used to inoculate 60 ml TY broth medium and grown on a gyratory shaker (200 rpm) at 28 °C until OD 0.6 was reached. DNA was isolated from 60 ml of cells using a CTAB bacterial genomic DNA isolation method [24]. The quality of DNA was checked by 0.5 % agarose gel electrophoresis and its quantity by a NanoDrop ND-1000 Spectrophotometer (Nano Drop Technologies, Wilmington, USA). A DNA concentration of 500 ng/μl and OD 260/OD 280 of 1.90 was obtained.

### Genome sequencing and assembly

The draft genome of *Bradyrhizobium* sp. WSM1253 was generated at the DOE Joint Genome Institute using a combination of Illumina [25] and 454 technologies [26]. For this genome, we constructed and sequenced an Illumina GAii shotgun library which generated 77,541,190 reads totaling 5,893.1 Mbp, a 454 Titanium paired end library with an average insert size of 12 Kbp which generated 615,580 reads totaling 123.4 Mbp of 454 data. All general aspects of library construction and sequencing performed at the JGI [27]. The initial draft assembly contained 274 contigs in 2 scaffolds. The 454 Titanium standard data and the 454 paired end data were assembled together with Newbler, version 2.3-PreRelease-6/30/2009. The Newbler consensus sequences were computationally shredded into 2 Kbp overlapping fake reads (shreds). Illumina sequencing data was assembled with VELVET, version 1.0.13 [28], and the consensus sequence was computationally shredded into 1.5 Kbp overlapping fake reads (shreds). We integrated the 454 Newbler consensus shreds, the Illumina VELVET consensus shreds and the read pairs in the 454 paired end library using parallel phrap, version SPS - 4.24 (High Performance Software, LLC). The software Consed [29–31] was used in the following finishing process. Illumina data was used to correct potential base errors and increase consensus quality using the software Polisher developed at JGI (Alla Lapidus, unpublished). Possible mis-assemblies were corrected using gapResolution (Cliff Han, unpublished), Dupfinisher [32], or sequencing cloned bridging PCR fragments with subcloning. Gaps between contigs were closed by editing in Consed, by PCR and by Bubble PCR (J-F Cheng, unpublished) primer walks. A total of 226 additional reactions were necessary to close gaps and to raise the quality of the finished sequence. The estimated genome size is 8.7 Mbp and the final assembly is based on 72.7 Mbp of 454 draft data which provides an average 8.4× coverage of the genome and 5,736.7 Mbp of Illumina draft data which provides an average 659.4× coverage of the genome.

### Genome annotation

Genes were identified using Prodigal [33], as part of the DOE-JGI genome annotation pipeline [34, 35] followed by a round of manual curation using GenePRIMP [36] for finished genomes and Draft genomes in fewer than 10 scaffolds. The predicted CDSs were translated and used to search the National Center for Biotechnology Information non-redundant database, UniProt, TIGRFam, Pfam, KEGG, COG, and InterPro databases. The tRNAScanSE tool [37] was used to find tRNA genes, whereas ribosomal RNA genes were found by searches against models of the ribosomal RNA genes built from SILVA [38]. Other non–coding RNAs such as the RNA components of the protein secretion complex and the RNase P were identified by searching the genome for the corresponding Rfam profiles using INFERNAL [39]. Additional gene prediction analysis and manual functional annotation was performed within the Integrated Microbial Genomes-Expert Review system [40] developed by the Joint Genome Institute, Walnut Creek, CA, USA.

## Genome properties

**Fig. 3:**
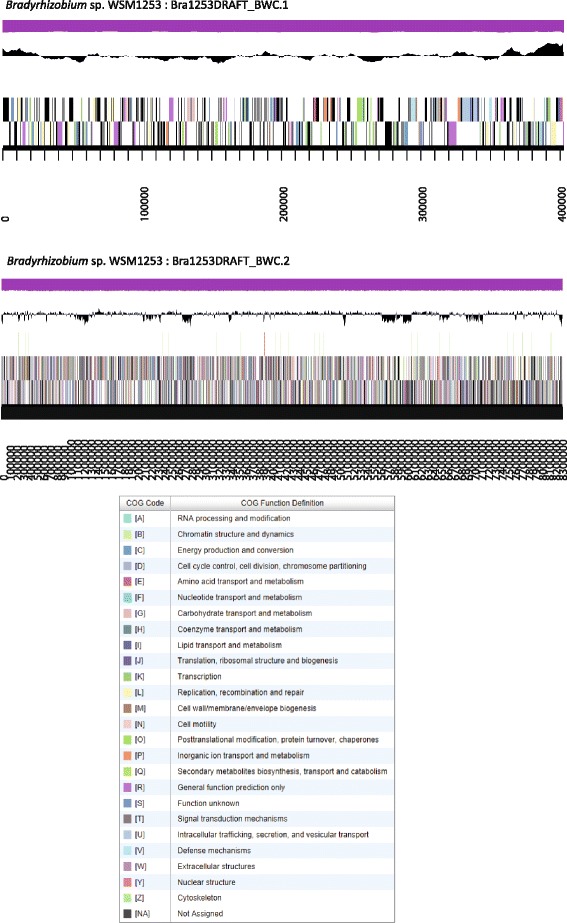
Graphical map of the two scaffolds from the genome of *Bradyhizobium* sp. WSM1253. From bottom to the top of each scaffold: Genes on forward strand (color by COG categories), Genes on reverse strand (color by COG categories), RNA genes (tRNAs green, sRNAs red, other RNAs black), GC content, GC skew

The genome is 8,719,808 nucleotides with 63.09 % GC content (Table 4) and comprised of 2 scaffolds (Fig. 3). From a total of 8,498 genes, 8,432 were protein encoding and 66 RNA only encoding genes. The majority of genes (66.86 %) were assigned a putative function whilst the remaining genes were annotated as hypothetical. The distribution of genes into COGs functional categories is presented in Table 5.

**Table 4:** Genome statistics for *Bradyhizobium* sp. WSM1253

Attribute	Value	% of Total
Genome size (bp)	8,719,808	100.00
DNA coding (bp)	7,446,464	85.40
DNA G + C (bp)	5,501,733	63.09
DNA scaffolds	2	100.00
Total genes	8,498	100.00
Protein coding genes	8,432	99.22
RNA genes	66	0.78
Pseudo genes	385	4.53
Genes in internal clusters	639	7.52
Genes with function prediction	5,682	66.89
Genes assigned to COGs	5,310	62.49
Genes with Pfam domains	6,484	76.30
Genes with signal peptides	948	11.16
Genes with transmembrane helices	1,953	22.98
CRISPR repeats	0	0.00

**Table 5:** Number of genes associated with general COG functional categories

Code	Value	% age	COG Category
J	235	3.83	Translation, ribosomal structure and biogenesis
A	0	0.00	RNA processing and modification
K	430	7.01	Transcription
L	1.53	2.50	Replication, recombination and repair
B	2	0.03	Chromatin structure and dynamics
D	39	0.64	Cell cycle control, cell division, chromosome partitioning
V	170	2.77	Defense mechanisms
T	270	4.40	Signal transduction mechanisms
M	322	5.25	Cell wall/membrane/envelope biogenesis
N	105	1.71	Cell motility
U	95	1.55	Intracellular trafficking, secretion, and vesicular transport
O	246	4.01	Posttranslational modification, protein turnover, chaperones
C	441	7.29	Energy production and conversion
G	418	6.82	Carbohydrate transport and metabolism
E	643	10.49	Amino acid transport and metabolism
F	94	1.53	Nucleotide transport and metabolism
H	322	5.25	Coenzyme transport and metabolism
I	387	6.31	Lipid transport and metabolism
P	361	5.89	Inorganic ion transport and metabolism
Q	261	4.26	Secondary metabolite biosynthesis, transport and catabolism
R	667	10.88	General function prediction only
S	360	5.87	Function unknown
-	3,188	37.51	Not in COGS

## Conclusions

*Bradyrhizobium* sp. WSM1253 was isolated from a nodule of the herbaceous annual legume *Ornithopus compressus* that was collected on the Greek Island of Sifnos. WSM1253 is rather unusual for a *Bradyrhizobium* strain in that it is highly efficient in nitrogen fixation for many species of *Lupinus* and *Ornithopus*, including *L. angustifolius, L. princei, L. atlanticus, L. pilosus, O. compressus, O. sativus* Brot. and *O. pinnatus*.

Phylogenetic analysis revealed that WSM1253 is most closely related to *Bradyrhizobium* sp. WSM1417. Strain WSM1417 was obtained from a *Lupinus* sp. nodule from Chile and differs from WSM1253 in that it cannot form an effective nitrogen-fixing symbiosis with *L. angustifolius*. The genomes of both of these strains have now been sequenced and this brings the total number of *Bradyrhizobium* genome depositions in IMG to 54; of these, strains which can symbiotically fix nitrogen have the nitrogenase-RXN MetaCyc pathway that is characterized by the multiprotein nitrogenase complex. However, strain WSM1253 is unique amongst these in that it can effectively fix nitrogen with many species of *Lupinus* (including *L. angustifolius, L. princei, L. atlanticus, L. pilosus*) and *Ornithopus compressus*. The genome attributes of *Bradyrhizobium* sp. WSM1253, in conjunction with other *Bradyrhizobium* genomes, will be important resources with which to build an understanding of interactions required for the successful establishment of effective symbioses with different species of *Lupinus* and *Ornithopus*.

## Additional file

Additional file 1: Table S1. Associated MIGS record. (DOC 73 kb)

## Abbreviations

½LA: half strength Lupin Agar
YMA: Yeast Mannitol Agar
TY: Tryptone Yeast extract Agar
GOLD: Genomes OnLine Database
CTAB: Cetyl trimethyl ammonium bromide
